# Microstructure, Thermal Stability, and Catalytic Activity of Compounds Formed in CaO-SiO_2_-Cr(NO_3_)_3_-H_2_O System

**DOI:** 10.3390/nano10071299

**Published:** 2020-07-02

**Authors:** Domante Niuniavaite, Kestutis Baltakys, Tadas Dambrauskas, Anatolijus Eisinas, Dovile Rubinaite, Andrius Jaskunas

**Affiliations:** 1 Department of Silicate Technology, Kaunas University of Technology, Radvilenu 19, LT-50270 Kaunas, Lithuania; domante.niuniavaite@ktu.edu (D.N.); tadas.dambrauskas@ktu.lt (T.D.); anatolijus.eisinas@ktu.lt (A.E.); dovile.rubinaite@ktu.edu (D.R.); 2 Department of Physical and Inorganic Chemistry, Kaunas University of Technology, Radvilenu 19, LT-50270 Kaunas, Lithuania; andrius.jaskunas@ktu.lt

**Keywords:** calcium chromate, calcium silicate, BET analysis, thermal stability, microstructure, calcium silicate hydrate, mesoporous, catalytic activity

## Abstract

In this work, the thermal stability, microstructure, and catalytic activity in oxidation reactions of calcium silicate hydrates formed in the CaO-SiO_2_-Cr(NO_3_)_3_-H_2_O system under hydrothermal conditions were examined in detail. Dry primary mixture with a molar ratio of CaO/SiO_2_ = 1.5 was mixed with Cr(NO_3_)_3_ solution (c = 10 g Cr^3+^/dm^3^) to reach a solution/solid ratio of the suspension of 10.0:1. Hydrothermal synthesis was carried out in unstirred suspensions at 175 °C for 16 h. It was determined that, after treatment, semicrystalline calcium silicate hydrates C-S-H(I) and/or C-S-H(II) with incorporated Cr^3+^ ions (100 mg/g) were formed. The results of in situ X-ray diffraction and simultaneous thermal analyses showed that the products were stable until 500 °C, while, at higher temperatures, they recrystallized to calcium chromate (CaCrO_4_, 550 °C) and wollastonite (800–850 °C). It was determined that both the surface area and the shape of the dominant pore changed during calcination. Propanol oxidation experiments showed that synthetic semicrystalline calcium silicate hydrates with intercalated chromium ions are able to exchange oxygen during the heterogeneous oxidation process. The obtained results were confirmed by XRD, STA, FT-IR, TEM, SEM, and BET methods, and by propanol oxidation experiments.

## 1. Introduction

According to various sources, humanity is facing more than 15 environmental concerns, such as air, water, and soil pollution, global warming, health issues, and others. One of the major contributors to global air pollution (denoted by the contribution to ozone and chemical smog) and human health is volatile organic compounds (VOCs, alkenes, alkanes, esters, alcohols, etc.) [1,2,3]. The main sources of these compounds are the chemical and petroleum industry, pharmaceutical plants, etc. In addition, a large variety of VOCs are generated from household products [4,5]. There are many technologies for VOC neutralization: Biological degradation, adsorption, ozonation, thermal treatment, and oxidation. Catalytic oxidation is one of the most common attractive ways to eliminate these compounds by converting them to CO_2_ and H_2_O at a low temperature (200–500 °C). The conventional catalysts require precious metals (Pt, Pd, Au), which are expensive; therefore, scientists are looking for new catalysts based on transitional metals [6,7,8,9].

In order to create a cost-effective and efficient catalyst for VOC oxidation, scientists are investigating such metals as Cr [10,11], Zn [12], Fe, Co [13,14,15], Cu [16,17], Mn [18], and others. One of the widely studied metals is chromium, which not only possesses efficient redox properties but is also economically attractive due to the low cost [19,20,21]. The application of chromium compounds for environmentally friendly selective oxidation reactions in the liquid or gas phases depends on the adhesion to specific catalyst supports. In addition, the nature of the catalyst support affects the performance, application, and properties of catalysts [22,23]. There are a lot of requirements for catalyst supports, but the high surface area and thermal stability are some of the most important ones. For this reason, attention has been paid to mesoporous calcium silicates (xCaO·ySiO_2_) or calcium silicate hydrates (xCaO·ySiO_2_·zH_2_O), which are a promising catalyst support [10,24]. These compounds not only have the aforementioned properties but are also chemically stable and able to disperse metal particles on the surface. In addition, calcium silicates and calcium silicate hydrates allow the retaining of the unique properties of metal ions and promote catalytic activity [25,26].

Calcium silicate hydrates form in nature, by curing cement, and they can also be synthesized in CaO-SiO_2_-H_2_O mixtures under hydrothermal conditions within the 100–350 °C temperature range [27]. Meanwhile, calcium silicates can be synthesized by calcining calcium silicate hydrates, or by solid sintering of calcium- and silicon-containing materials [28,29]. Usually, the preparation of catalysts supported with calcium silicates or calcium silicate hydrates involves three steps [30,31]: (1) Synthesis of calcium silicates or calcium silicate hydrates; (2) adsorption of metal ions; (3) calcination at a selected temperature (in order to achieve active metal oxides). In addition, it is possible to incorporate metal ions into the structure of calcium silicates or calcium silicate hydrates during their synthesis. Such compounds with incorporated metal ions can be used as catalysts for ethanol conversion to butadiene, for the synthesis of bisphenol F, and for the oxidation of ketones and aldehydes [25,32,33]. Unfortunately, there is a lack of information about the influence of metals on the formation, thermal stability, microstructure, and other properties of such materials. According to the literature, some metal ions, such as sodium or potassium ions, have a positive effect on the reactivity of silicon-containing compounds and promote the formation of calcium silicate hydrates [34]. Meanwhile, aluminum ions affect the stability and morphology of tobermorite because, by increasing the aluminum content, the form of crystals changes from plate-like to lath-like and then to needle-like [35]. Different results were obtained by using aluminum oxide for the synthesis of dibasic calcium silicate hydrate α-C_2_SH at 200 °C because this additive retarded the formation of calcium silicate hydrates but stimulated the crystallization of calcium silicate [28]. According to the literature, some cations, such as Al^3+^, B^3+^, and Be^2+^, can change silicon in the silicon–oxygen tetrahedron, while others (Na^+^, K^+^, Fe^2+^, Mn^2+^, Ti^2+^, Zr^2+^, etc.) intercalate outside it. These cations connect silicon–oxygen tetrahedrons to each other. Thus, different cations change the composition, structure, and other properties of calcium silicate hydrates and calcium silicates [36].

For these reasons, in the first part of this work, the thermal stability of compounds formed in the CaO-SiO_2_-Cr(NO_3_)_3_-H_2_O system under hydrothermal conditions was examined in detail. Meanwhile, in the second part, the microstructure and catalytic activity of synthetic and calcined products were determined.

## 2. Materials and Methods

The synthesis of dibasic calcium silicate hydrate samples (C-S-H) with incorporated Cr^3+^ ions was based on the hydrothermal method. The dry primary mixture of fine-grained SiO_2_·nH_2_O (Reaktiv, Saint Petersburg, Russia, loss of ignition—16.9%) and calcium oxide (produced by burning Ca(OH)_2_ (Sigma Aldrich, Darmstadt, Germany) at 450 °C for 1 h; the quantity of free CaO was equal to 97.41%) was mixed with CrN_3_O_9_·9H_2_O solution (Eurochemicals, Vilnius, Lithuania, concentration of Cr^3+^ ions 10 g/dm^3^) to reach a water-to-solid ratio of 10:1, with the CaO/SiO_2_ molar ratio of 1.5:1. Due to the formation peculiarities of higher-basicity calcium silicates hydrates [27,28,30,37], hydrothermal synthesis was carried out in unstirred suspensions under saturated steam pressure at 175 °C for 16 h. After hydrothermal treatment, the obtained products were filtered off, rinsed with ethanol, dried at 50 °C ± 5 for 24 h, and sieved (<80 μm).

The obtained products were calcined in a high-temperature furnace Nabertherm LH 15/13 at 550 °C for 24 h. The calcination temperature was reached within 48 h.

The mineralogical composition of products was determined by powder X-ray diffraction (XRD, D8 Advance diffractometer, Bruker AXS, Karlsruhe, Germany). The operating conditions were as follows: 0.02 mm Ni filter, Cu Kα radiation, tube voltage 40 kV, tube current 40 mA, detector Bruker LynxEye. Diffraction patterns were recorded in a Bragg–Brentano geometry within the 2*θ* range of 3–70° at a scanning speed of 6°/min.

The measurements of the thermal stability and phase transformation were prepared with:

(1) Linseis PT1000 instrument (Linseis, Selb, Germany). The operating conditions were: A heating rate of 15 °C/min, temperature range of 30–1000 °C, nitrogen atmosphere, ceramic sample handlers, and crucibles of Pt, and the sample mass was equal to ~13 mg.
(2) In-situ XRD analysis was made with a high-temperature camera MTC-hightemp (Bruker AXS, Karlsruhe, Germany). The measurements were carried out with a step width of 0.02 2*θ* and 0.6 s/step at a heating rate of 50 °C/min after equilibration for 5 min at the desired temperature.

Fourier-transform infrared spectroscopy was carried out with the help of a Perkin Elmer FT–IR Spectrum X system (PerkinElmer, Waltham, MA, USA). Specimens were prepared by mixing 1 mg of the sample with 200 mg of KBr. Spectral analysis was performed in the range of 4000–400 cm^−1^ with a spectral resolution of 1 cm^−1^.

The microstructure of the products was determined by using:

(1) Scanning electron microscopy was performed by using a JEOL JSM-7600F (JEOL, Tokyo, Japan) instrument at an accelerating voltage of 10 kV, and a working distance of 8.6 mm.
(2) Transmission electron microscopy was performed by using a Tecnai G2 F20 X-TWIN instrument (FEI, Eindhoven, The Netherlands) with a Schottky-type field-emission electron source. The accelerating voltage was 200 kV.

The concentration of Cr^3+^ ions was determined by using a Perkin-Elmer Analyst 400 atomic absorption spectrometer (Perkin Elmer, Waltham, MA, USA) with the following parameters: Wavelength = 357.87 nm; hollow cathode lamp current (I) = 30 mA; the type of flame was C_2_H_2_–air; oxidant air = 10 L/min; acetylene = 2.5 L/min. All the tests were repeated three times. The concentration of nitrate anions was determined by using a Flow Injection Analyst FIAlyzer-100 (FIA; FIAlab Instruments, Seattle, WA, USA). FIAlyzer-100 system: FIAlyzer-100, integrated FIA LOB manifold, USB4000 UV/VIS spectrometer, HL2000-LL visible tungsten lamp.

The surface area of the samples was measured with a BET surface area analyzer (nitrogen adsorption porosimeter) Nova 2200 E-Series (Quantachrome Instruments, Boynton Beach, FL, USA). Prior to analysis, the samples were degassed under vacuum at 100 °C. The specific surface area of the samples was calculated with the BET equation by using the data of the lower part of the N_2_ adsorption isotherm (0.05 < *P/P_0_* < 0.35). The total pore volume, the pore size distribution, and the shape of pores were calculated according to the corrected Kelvin equation and the scheme developed by Orr et al. by using the entire N_2_ desorption isotherm at 77 K [38,39].

The catalyst activity in oxidation reactions was determined as follows. Propanol was used as a volatile organic compound for catalytic oxidation experiments. In addition, 0.945–1.009 g of the analyzed sample was placed inside a fixed-bed quartz reactor equipped with a coil preheater. A quartz reactor was mounted inside a Nabertherm LH 15/13 furnace for maintaining the constant temperature, while a K-type thermocouple inside the reactor was used for accurate temperature monitoring. The inlet and the outlet of the reactor are equipped with special analysis points for the collection of gaseous flow samples, as well as CO and CO_2_ concentration measurement probes connected directly to a TESTO 445 unit. Catalytic oxidation was performed with a constant 370 mL/min flow of air, which was saturated with 475–640 ppm of VOC. The concentrations of propanol in the gas stream were determined with a Perkin Elmer Clarus 500 GC/MS system equipped with a COL-ELITE 5MS universal capillary column, which is 30 m long and has a 0.25 mm internal diameter.

## 3. Results and Discussions

### 3.1. Synthesis of Calcium Silicate Hydrates with Incorporated Cr^3+^ Ions

**Figure 1:**
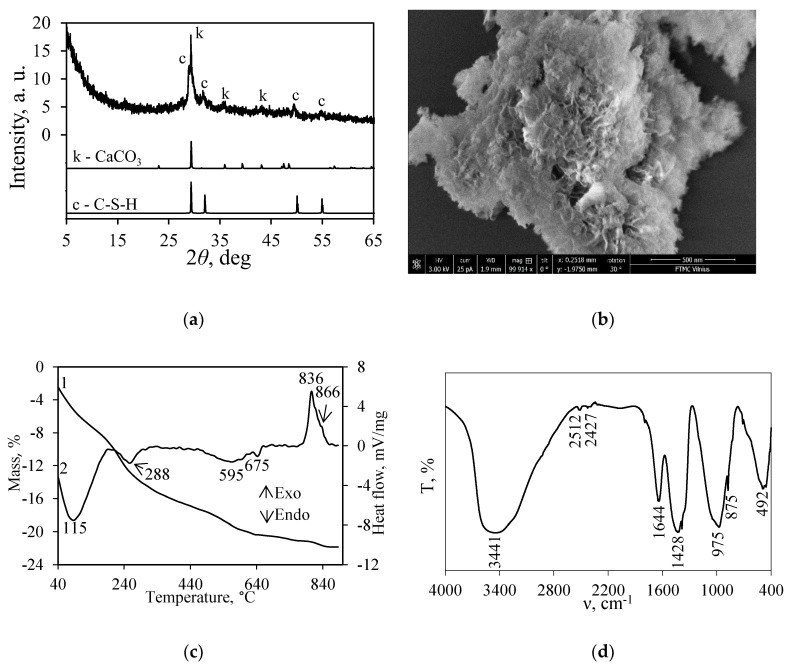
XRD pattern (**a**), SEM image (**b**), STA curves (curve 1—TG; curve 2—DSC) (**c**), and FT-IR spectrum (**d**) of the synthesis products. Indexes: k–calcite, c–C-S-H(I)/C-S-H(II).

The data of X-ray diffraction analysis showed that, during the hydrothermal treatment in the CaO-SiO_2_-Cr(NO_3_)_3_-H_2_O mixture at 175 °C, semicrystalline calcium silicate hydrates C-S-H(I) and/or C-S-H(II) (*d*-spacing—0.303; 0.280; 0.184; 0.167 nm) were formed in the products (Figure 1a) [38,39]. In addition, due to the carbonization when the products were dried in the air-conditioned chamber, traces of calcite (PDF No. 04-012-0489) were detected in the XRD pattern [40]. It is worth mentioning that chromium ions do not affect the mineral composition of the synthesis products because, under the same conditions of synthesis in the pure system (CaO-SiO_2_-H_2_O), only C-S-H(I), C-S-H(II), and calcite were formed [30].

The results of atomic absorption spectroscopy showed that, after synthesis, the concentration of Cr^3+^ ions in the liquid medium decreased by more than 99.99%, i.e., from 10,000 mg/dm^3^ (before synthesis) to 0.041 mg/dm^3^ (after synthesis) (Supplementary Materials, Table S1). Meanwhile, the results of FIA analysis showed that more than 80% (from the primary amount of 17,308 mg/dm^3^) of the NO_3_^–^ anions are present in the liquid medium (Supplementary Materials, Table S2). It should be noted that the XRD results did not show the formation of crystalline compounds containing Cr^3+^ or NO_3_^−^ ions. Thus, it can be stated that all Cr^3+^ ions intercalated into the structure of calcium silicate hydrates or formed amorphous compounds, while NO_3_^−^ anions only partially (−20%) participated in the process. These data are in good agreement with the results obtained in previous works [30,41], which determined that synthetic calcium silicate hydrates act as a chemo-sorbent, and their adsorption capacity is equal to 100 mg Cr^3+^/1 g CSH.

In the SEM micrograph of the synthesis products (Figure 1b), only thin close-packed particles (called foils or honeycomb) characteristic to C-S-H(I) and C-S-H(II) were observed [38,42]. In addition, the existence of the above-mentioned compounds was confirmed by DSC data: The endothermic effect at 116 °C can be assigned to the removal of adsorption/crystallization water in semicrystalline calcium silicate hydrates, while the exothermic effect at 836 °C and the shoulder at 866 °C are characteristic of the recrystallization process of C-S-H(I) and C-S-H(II), respectively (Figure 1c, curve 2). Furthermore, the endothermic effects at 288 °C and 595 °C can be assigned to the dehydration or decomposition of compounds containing Cr^3+^ and/or NO_3_^–^ ions, as well as to the formation of double metal oxides (Figure 1c, curve 2) [43]. The small thermal effect at 675 °C corresponds to the decomposition of calcium carbonate. The data of TGA showed that less than 0.5% of the above-mentioned compound is present in the products (Figure 1c, curve 1).

The identification of the absorption bands in the FT-IR spectrum of the synthesis products is complicated (Figure 1d). The adsorption band, which is present within the 400–700 cm^−1^ frequency range, is typical to semicrystalline C-S-H (*δ*(Si–O–Si) and *δ*(O–Si–O)) [44] and/or to *ν*(Cr–O) vibrations [45]. In a higher frequency interval (800–1000 cm^−1^), the absorption bands can be assigned to symmetrical *ν_s_*(O-SiO-) vibrations in the C-S-H structure [46]. Furthermore, the adsorption maximums at –1428 and –875 cm^−1^ correspond to *ν*(CO_3_^2−^) and *δ*(C–O_3_^2−^) or NO_3_^−^ group vibrations, respectively [47]. Meanwhile, the adsorption band at 1644 cm^−1^ can be assigned to the vibration of the OH^−^ bonds in both C-S-H and compounds containing the Cr^3+^ ions structure. Finally, the broad band within the 2500–4000 cm^−1^ range reflected the H-O-H bending vibration of water. It is worth mentioning that the adsorption bands associated with the NO_3_^−^ group occur in the same frequency interval as *ν*(CO_3_^2−^) and OH^−^ vibrations.

### 3.2. Thermal Stability of Synthesis Products

**Figure 2:**
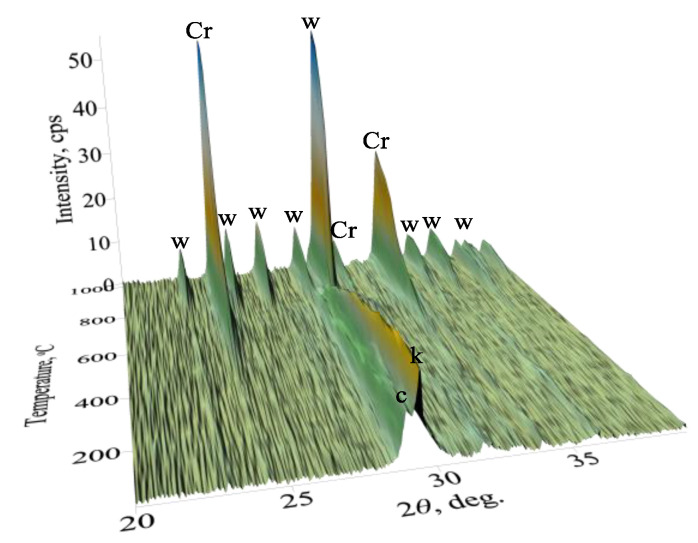
In-situ XRD patterns of synthesis products when the temperature of calcination is 25–1000 °C. Indexes: c—C-S-H(I)/C-S-H(II); w—wollastonite; k—CaCO_3_; Cr—CaCrO_4_.

In order to determine the formation of the potentially catalytic active compounds, the synthesis products were calcined in a high-temperature camera MTC-hightemp within the 25–1000 °C temperature range (Figure 2). Calcination was carried out at a heating rate of 50 °C/min after equilibration for 5 min at the desired temperature. The results of in-situ XRD patterns showed that the synthesis products are stable within the 25–550 °C temperature range (Figure 2). It should be noted that the DSC curve showed two endothermic effects at 115 °C and 288 °C temperatures (Figure 1c). The difference between the presently mentioned results may have been observed due to the dehydration and/or decomposition of the amorphous phase, which cannot be identified in XRD patterns. By increasing the calcination temperature (>550 °C), the formation of calcium chromate CaCrO_4_ (PDF 00-008-0458) proceeded (Figure 2). According to the literature, this compound can be used for scintillation, Raman scattering behavior, or as a catalyst, dielectric material, paint pigment, and lubricant, and for wastewater treatment [48,49,50,51]. It was determined that calcium chromate remained stable until 1000 °C (Figure 2). These data are in good agreement with DSC results (Figure 1c). In addition, when the calcination temperature was increased to 800–850 °C, the formation of calcium silicate—wollastonite (PDF 00-066-0271, Supplementary Materials, Figure S1)—was observed (Figure 2).

**Figure 3:**
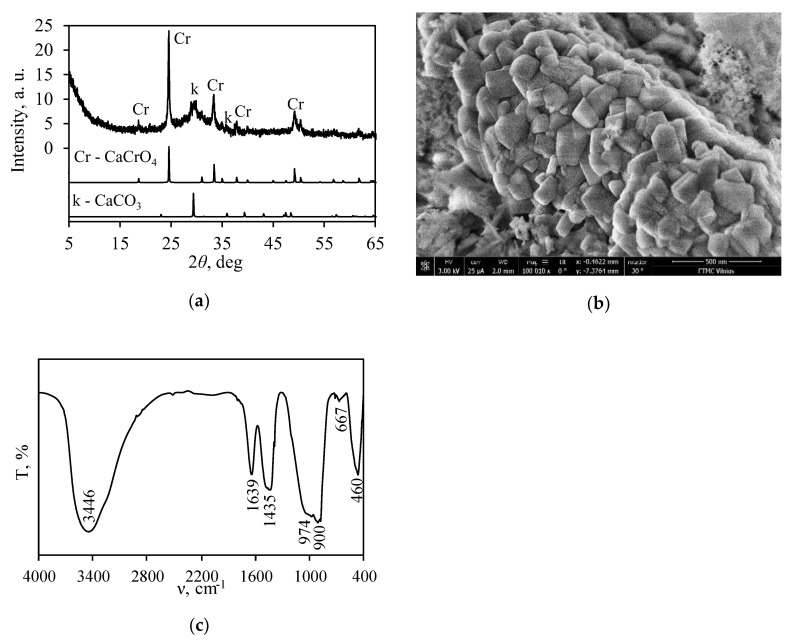
XRD pattern (**a**), SEM image (**b**), and FT-IR spectrum (**c**) of the calcined sample at 550 °C. Indexes: k—CaCO_3_; Cr—CaCrO_4_.

In order to obtain a calcined sample with calcium chromate and a high surface area, a synthetic sample was calcined in the furnace Nabertherm LH 15/13 at 550 °C for 24 h. The calcination temperature was reached within 48 h. It was determined that, after calcination, only one crystalline compound—calcium chromate—was formed (Figure 3a). In addition, a broad basal reflection within the 25–37° 2*θ* range can be assigned to partially dehydrated semicrystalline calcium silicate hydrates. It is worth mentioning that, under these conditions of calcination, the traces of calcite remained stable.

The results of SEM analysis are in good agreement with the data of XRD (Figure 3a) because small crystals typical of CaCrO_4_ were observed (Figure 3b). In addition, in the FT-IR spectrum, the intensive adsorption band at 900 cm^–1^ can be attributed to the vibrations of Cr^+6^–O bonds in CaCrO_4_ [45] (Figure 3c). It should also be observed that, under these conditions of calcination, the dehydration of the semicrystalline-type compounds proceeded partially because intensive bands characteristic of OH^−^ group vibrations in the FT-IR spectrum are visible (Figure 3c). These results are in good agreement with TGA results because the mass changes of the synthetic product in the 550–900 °C temperature range are equal to 4.14% (Figure 1c). In addition, similar results were presented in the literature [52].

### 3.3. Porosity of Synthetic and Calcined Products

It is known that one of the most important parameters of all catalysts and adsorbents is a high surface area, which leads to successful application [38,53,54]. Thus, in order to determine the specific surface area, nitrogen gas adsorption in combination with the Brunauer, Emmett, and Teller (BET) equation was performed. Meanwhile, the shape of meso- and macro-pores and their distribution were calculated by using the corrected Kelvin equation and the scheme as developed by Orr et al.

**Figure 4:**
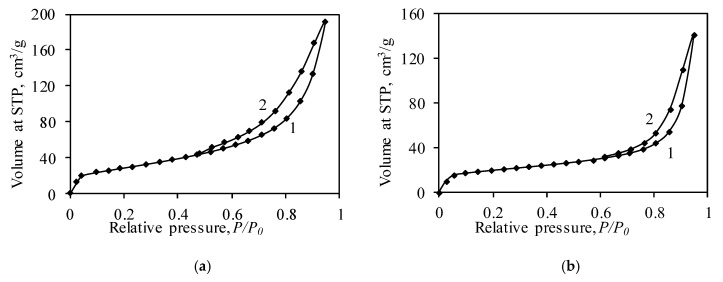
Adsorption (1)–desorption (2) isotherms of synthetic (**a**) and calcined (**b**) samples.

The nitrogen adsorption–desorption isotherms of the samples are presented in Figure 4. According to the IUPAC classification [55], the adsorption isotherms of both samples can be classified as Type IV whose characteristic feature is a hysteresis loop. According to the literature, this type of isotherm should feature a plateau at high *P/P*_0_ values (>0.7), which indicates complete pore filling. In the case of synthetic and calcined samples, the plateau was not present in the adsorption/desorption isotherms (Figure 4). This phenomenon can be explained by unrestricted multilayer adsorption on the surfaces of samples. Similar adsorption–desorption isotherms were obtained by Naderi [56], and Babaee and Castel [57]. According to Rouquerol [58] and Sun [59], the Type IV isotherm is characteristic of mesoporous materials (the pore diameter varies within the 2–50 nm range), where the formation of a monolayer and a multilayer does not overlap; thus, the BET equation can be used for the characterization of materials.

Further analysis of the isotherms showed that the hysteresis loop of the synthetic product corresponds to Type H1 and/or Type H3 (Figure 4a). The H1 hysteresis loop is characteristic of mesoporous materials consisting of spherical particles or possessing well-defined cylindrical-like pore channels [55,60]. Usually, materials with a H1 hysteresis loop have connected similarly sized pores. Meanwhile, the H3 hysteresis loop is detected in materials consisting of plate-like particles and slit-like pores. This type of hysteresis loop does not have limiting adsorption at high *P/P*_0_ and closes in the range of *P/P*_0_ 0.4–0.45. Thus, it possible that, in the synthetic product, both cylindrical-like and slit-like pores were formed (Figure 4). These data are in good agreement with the SEM results (Figure 1b) and the literature data. According to Liu et al. [31] and Zhang [61], during the hydrothermal synthesis of calcium silicates hydrates, cylindrical-like, slit-like, or ink-bottle pores may be present in the products.

It was determined that, during calcination, the shape of the dominant pores changed from a mixture of cylindrical-like and slit-like pores to well-defined cylindrical-like pores (Figure 4). The changes may have occurred due to solid sintering reactions and the formation of new compounds (with a different crystal lattice) during calcination (Figure 2). According to the literature, the changes in the pore shape have an influence on the surface area, the pore size distribution, and the physical-chemical properties of the catalyst, adsorbents, and membranes. Feinberg et al. [60] determined that membranes with slit-like pores have a higher selectivity than membranes with cylindrical-like pores. In addition, the accessibility of the active sites of the catalyst, as well as the surfaces of adsorbents, depends on the structure of the pores, their diameter, and the pore network.

**Figure 5:**
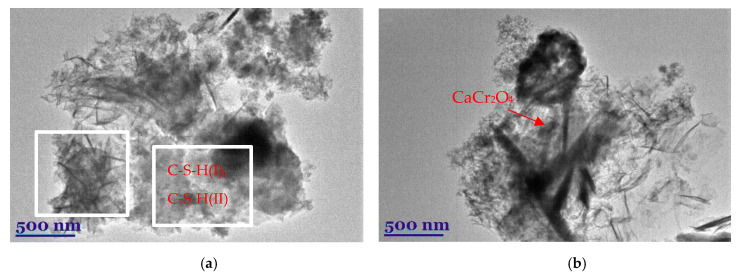
TEM micrographs of synthetic (**a**) and calcined (**b**) samples.

The presently discussed results were confirmed by TEM data (Figure 5). The TEM micrograph of the synthetic C-S-H sample with chrome ions showed an amorphous mass and needle-like crystallites (Figure 5a). Presumably, cylindrical-like pores form in amorphous mass, while slit-like pores form between needle-like crystallites. Meanwhile, in a calcined sample, compounds of the amorphous structure (partially dehydrated C-S-H(I) and C-S-H(II)) and plate-like crystallite of CaCr_2_O_4_ [62] were identified (Figure 5b).

**Figure 6:**
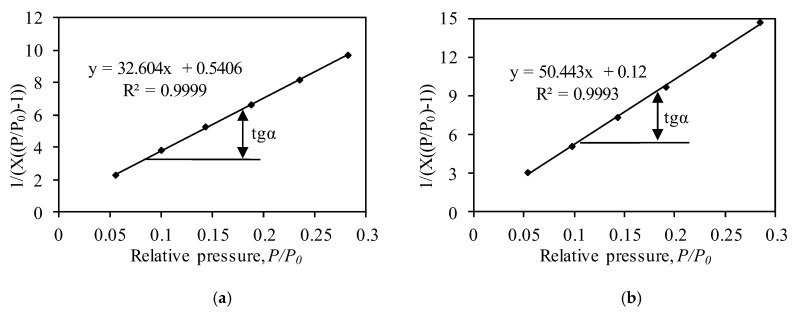
Isotherm of N_2_ adsorption at 77 K in BET plot of synthetic (**a**) and calcined (**b**) samples.

The BET method was used for the calculation of the surface area of the samples (Figure 6, Table 1), which affects such properties of materials as their dissolution rate, adsorption capacity, etc. [63,64]. According to the literature [56,58], the BET method is valid if a straight line in the BET coordination (1/[*X*((*P*_0_/*P*) − 1)]) − (*P*/*P*_0_) is obtained (Figure 6), and the value of the C_BET_ constant is higher than 2 (in the ideal case, it is between 50 and 300). Calculations revealed that the values of the C_BET_ constant of synthetic and calcined samples are equal to 61.31 and 421.30, respectively. The higher value of the C_BET_ constant of the calcined sample can be explained by nitrogen adsorption on high-energy surface sites (probably CaCr_2_O_4_ crystals) or the filling of micropores [55,65]. It was calculated that the synthetic sample has a relatively large surface area of 105.14 m^2^/g (Table 1). It is worth noting that the S_BET_ value of synthetic calcium silicates hydrates usually varies within the 30–500 m^2^/g range [66,67]. It was determined that, during calcination, at 550 °C, the value of the surface area decreased to 68.92 m^2^/g (Table 1). The decreases in the surface area can be related to the changes in the pore shape from cylindrical-like and slit-like pores (after synthesis) to well-defined cylindrical-like pores (after calcination) (Figure 4). Although the value of the surface area decreased, it is still 2–20 times higher compared to other calcium silicates: Wollastonite−2 m^2^/g, kilchoanite−6 m^2^/g, rankinite−11.7 m^2^/g, and others [38,68,69]. Meanwhile, the surface area of the catalyst depends on the starting raw materials, as well as on the synthesis conditions, and it can vary within the 13–1000 m^2^/g range [70].

The aforementioned analysis of adsorption isotherms and the visual inspection of the shape of the pores (TEM, SEM) are fairly complicated; thus, in order to confirm the obtained results and to calculate the dominating pore size, the cumulative pore volume, the corrected Kelvin equation, and the scheme developed by Orr et al. were applied. The calculations were done by using models of cylindrical-like and slit-like pores formed between parallel plates. The employed model is valid as long as the difference between S_BET_ and ∑A is not significant, i.e., it does not exceed 10%. The calculations of the dominant pore shape in the structure of synthetic samples showed a fairly significant difference between the S_BET_ and ∑A values (Table 2). These results confirm the hysteresis loop classification as an intermediate case between H1 and H3 (Figure 4a). In addition, it can be stated that both cylindrical-like and slit-like pores were formed in the structure of the synthetic sample. Further calculations showed that, during calcination, the shape of the pores changed to a well-defined cylindrical one because the difference between the S_BET_ and ∑A values was equal to only 2.87% (Table 2).

**Figure 7:**
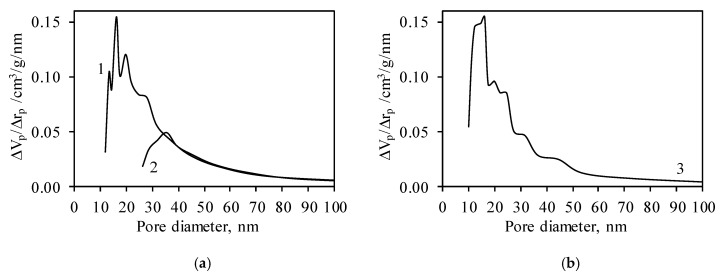
Differential distributions of pore sizes of synthetic (**a**) and calcined (**b**) samples. Here, curve 1 and curve 3 values were obtained by using the model of cylindrical-like pores, and curve 2 values were obtained by using the model of slit-like pores.

It was calculated that the value of the cumulative pore volume of the synthetic sample depends on the employed model and is equal to 0.278 and 0.320 cm^3^/g (Table 2). Differential distributions of the pore sizes showed that the synthetic sample is a mesoporous material because the pores with the 10–60 nm diameter are dominant (Figure 7a). It is worth mentioning that the employed model has a significant influence on the distributions of the pore sizes (Figure 7a). Unfortunately, both types of pores were present in the structure of the synthetic samples, and, as a result, the right distribution is arguable.

It was determined that, during calcination, the cumulative pore volume of the sample decreased to 0.230 cm^3^/g (Table 2). The obtained value is sufficient for the preparation of adsorbents or catalysts because the cumulative pore volume of zeolites and aluminum oxide varies within the 0.2–0.4 cm^3^/g range [70,71]. Meanwhile, the results of differential distributions of the pore sizes showed that the calcined sample is a mesoporous material with dominant 10–30 nm pores (Figure 7b). In addition, in the structure of the calcined sample, pores of a higher diameter (30–60 nm) are present.

### 3.4. Catalyst Activity in Oxidation Reactions

The catalytic activity of synthesized and calcined samples was measured during complete oxidation of propanol in an air stream. The main product of complete oxidation is carbon dioxide; thus, the main parameter for catalyst performance is the decrease in the concentration of the volatile organic compound in relation to CO_2_ accumulation. As the initial concentration of propanol slightly varied, all the measured concentration values were normalized and are presented as one gram of catalyst per one gram of propanol in the ingoing stream. The decrease in the propanol concentration is presented as conversion to percentage units, whereas the selectivity of the catalysts was evaluated by the amounts of intermediates found in the outgoing flow. Experiments were performed within the temperature range of 150–300 °C with the step of the temperature increasing by around 25 °C per hour.

**Figure 8:**
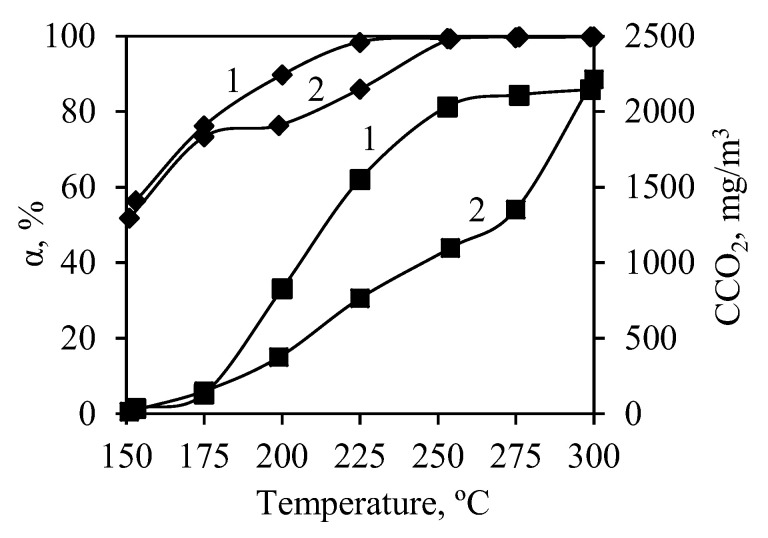
Degree of conversion (♦) and accumulated CO_2_ concentration (■) during catalytic oxidation of propanol in synthetic (1) and calcined (2) samples.

It was determined that, at the beginning of the experiments (150 °C), by using synthetic and calcined samples, the concentration of propanol in the outgoing stream decreased to 56.5% and to 51.8%, respectively (Figure 8). However, further analysis showed the absence of CO_2_ in the outgoing stream, which indicates that propanol was adsorbed by samples but was not oxidized. The higher adsorption of propanol by the synthetic sample can be explained by its higher surface area (105.14 m^2^/g) than that of the calcined sample (68.92 m^2^/g) (Table 1).

Propanol oxidation starts at a temperature of 175 °C because the CO_2_ concentration increases in the outgoing stream. At this temperature, the differences between synthetic and calcined samples appear. The synthetic sample showed a higher catalytic activity than the calcined one because a sharper increase in CO_2_ concentration was observed (Figure 8). Conversion and CO_2_ accumulation curves coincide well, which indicates that, with the increase in temperature, the process switches from adsorption toward catalytic oxidation.

By comparing the aforementioned curves, it can be stated that the synthetic sample reached 95% conversion at around 240 °C, which is a good result comparable to bulk, supported, and mixed catalysts [53,54,72]. This indicates that semicrystalline calcium silicate hydrates with intercalated chromium ions are able to exchange oxygen during the heterogeneous oxidation process. It should be noted that chlorinated organics, as well as esters, are less destructible than alcohols. It was determined that the calcined sample struggled with catalytic oxidation as the formation of carbon dioxide was much slower—conversion of 95% was reached only at temperatures higher than 290 °C (Figure 8). It can be concluded that the formation of calcium chromate has a negative effect on propanol oxidation reactions; thus, the synthetic sample should be used at lower temperatures than 500 °C (in order to avoid the formation of calcium chromate). Similar results are presented in the literature, specifically that copper dichromate did not show high catalytic activity [72]. Evidently, both samples had high adsorptive affinity for propanol, as the influence of adsorption on the overall process was observed at temperatures as high as 275 °C. As the temperature was increased in the catalyst bed, sharp increases in CO_2_ concentration, as well as sudden spikes in temperature due to the exothermal effect of the oxidation reaction, were observed.

**Figure 9:**
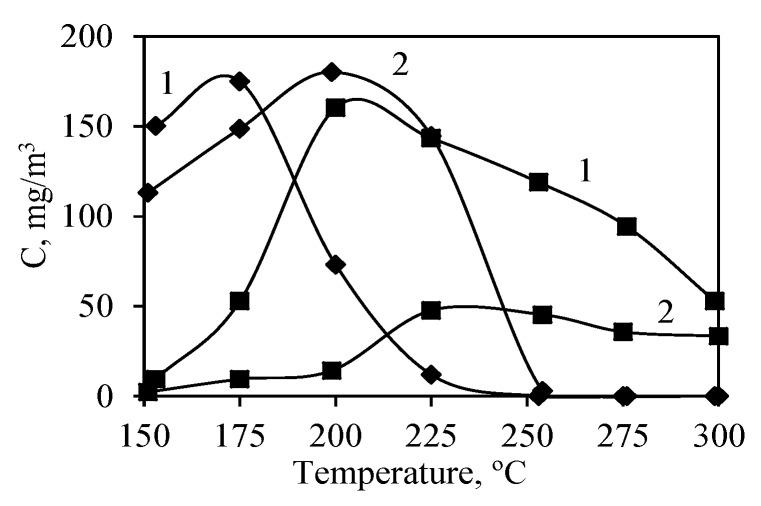
Changes in the concentrations of pentanal (♦) and CO (■) formed during propanol catalytic oxidation in synthetic (1) and calcined (2) samples.

The CO probe and GC/MS monitoring of the outgoing gas stream showed the appearance of incomplete catalytic oxidation product intermediates. Usually, carbon monoxide forms at the beginning of catalytic oxidation reactions, and it is oxidized to CO_2_ faster by increasing the temperature. In this case, the formation of CO could also be used for the determination of catalytic activity. The initial formation of CO could be observed at lower temperatures only for the synthetic sample, where it reached the maximum value of 161 mg/m^3^ at 200 °C and started decreasing afterward (Figure 9). Meanwhile, the calcined sample reached the maximum concentration of CO (48 mg/m^3^) at a temperature higher by 25°, i.e., at 225 °C, and it was more than three times lower in comparison to the synthetic sample. CO formation curves (Figure 9) showed that the lower formation of carbon monoxide during oxidation on the calcined sample is caused by the lower overall activity, but not due to higher selectivity.

Both samples showed similar results for the formation of the second intermediate compound—propanal—where they yielded similar amounts of this aldehyde, –175 mg/m^3^ for the synthesized sample and 180 mg/m^3^ for the calcined sample (Figure 9). The formation of propanal takes place up until catalytic oxidation takes over adsorption, and the maximum values are reached at 175 °C for the synthesized sample and at 200 °C for the calcined sample. Although aldehydes are harder to oxidize than alcohols, because of the relatively smaller amount of propanal in the stream, its concentration still decreases fast as the temperature in the catalyst bed increases, and it can no longer be detected at 250 °C. Propanal forms as a result of the interaction between propanol and the surface of the catalyst, which means dehydrogenation of propanol takes place. Therefore, it is evident that the largest amount of propanal forms when the adsorption process is the strongest, and it is also attributed to the apparent decrease in the propanol concentration before the start of catalytic oxidation reactions.

## 4. Conclusions

It was determined that, after 16 h of hydrothermal treatment at 175 °C, semicrystalline calcium silicate hydrates C-S-H(I) and/or C-S-H(II) were formed in the products. The analysis of the liquid medium showed that all Cr^3+^ ions intercalated into the structure of the synthesis products or formed amorphous compounds, while NO_3_^–^ anions only partially (~20%) participated in the process.

It was determined that, during calcination at 550 °C, the formation of calcium chromate (CaCrO_4_) proceeded, and it remained stable until 1000 °C. The formation of this compound was confirmed by SEM and FT-IR analysis: In SEM micrographs, crystals characteristic of CaCrO_4_ were observed, while, in the FT-IR spectrum, an intensive adsorption band at 900 cm^–1^ of Cr^+6^–O vibrations was identified.

It was determined that both synthetic and calcined (550 °C) samples are mesoporous materials whose specific surface area (S_BET_) is equal to 104.76 and 68.92 m^2^/g, respectively. It was determined that cylindrical-like and slit-like pores are present in the structure of the synthetic samples, while, during calcination at a temperature of 550 °C, the shape of the pores changed to well-defined cylindrical-like pores. These data were confirmed by SEM, as well as TEM results, and by calculations using the corrected Kelvin equation and the Orr et al.-developed scheme.

It was determined that at temperatures lower than 175 °C, synthetic and calcined samples act as adsorbents, while, at higher temperatures, catalytic oxidation proceeds. It was determined that the synthetic sample reached 95% conversion at around 240 °C, which is a good result, while the calcined sample reached this value only at temperatures higher than 290 °C. It can thus be concluded that the formation of calcium chromate has a negative effect on propanol oxidation reactions; therefore, the synthetic sample should be used at temperatures lower than 500 °C.

## Figures and Tables

**Table 1:** Calculated parameters of synthetic and calcined samples by BET method.

Sample	BET Equation Constants		Capacity of Mono Layer Xm	S_BET_, m^2^/g	C_BET_ Constant	Reliability Coefficient, R^2^
	Slope/S = tgα	Intercept/*I*				
Synthetic	32.76	0.51	0.030	104.76	65.56	0.995
Calcined	50.44	0.12	0.020	68.92	421.30	0.995

**Table 2:** Data of ΣA and ΣV_P_ calculations of samples.

Sample	S_BET_, m^2^/g	Results Obtained by Using the Model of Cylindrical Pores				Results Obtained by Using the Model of Slit-Like Pores			
		ΣA, m^2^/g	|S_BET_ − ΣA|, m^2^/g	|S_BET_ − ΣA|, %	ΣV_P_, cm^3^/g	ΣA, m^2^/g	|S_BET_ − ΣA|, m^2^/g	|S_BET_ − ΣA|, %	ΣV_P_, cm^3^/g
Synthetic	105.14	134.46	29.32	27.89	0.320	84.46	20.68	19.67	0.278
Calcined	68.92	66.94	1.98	2.87	0.230	37.27	31.65	45.92	-

## Data Availability

The data supporting the findings of this study are available by request from the corresponding author: Tadas Dambrauskas (tadas.dambrauskas@ktu.lt).

## Supplementary Materials

The following are available online at https://www.mdpi.com/2079-4991/10/7/1299/s1, Figure S1: XRD pattern of wollastonite (PDF 00-066-0271), Table S1: Concentration of Cr^3+^ ions in the liquid medium, Table S2: Concentration of NO_3_^−^ ions in the liquid medium.
