# Supplementary material for: Microstructure, Thermal Stability, and Catalytic Activity of Compounds Formed in CaO-SiO2-Cr(NO3)3-H2O System
Source: Nanomaterials (Basel). 2020 Jul 2;10(7):1299. doi: 10.3390/nano10071299 (PMC7407582; doi:10.3390/nano10071299)
Supplement: Supplementary file 1 [file nanomaterials-10-01299-s001.pdf]

nanomaterials

Supplementary Materials

Microstructure, Thermal Stability, and Catalytic Activity of Compounds Formed in CaO-SiO_2-$Cr(NO_3)_3$-H_2O System

Domante Niuniavaite [1], Kestutis Baltakys [1,*], Tadas Dambrauskas [1], Anatolijus Eisinas [1], Dovile Rubinaite [1] and Andrius Jaskunas [2]

[1] Department of Silicate Technology, Kaunas University of Technology, Radvilenu 19, LT-50270 Kaunas, Lithuania; domante.niuniavaite@ktu.edu (D.N.); tadas.dambrauskas@ktu.lt (T.D.); anatolijus.eisinas@ktu.lt (A.E.); dovile.rubinaite@ktu.edu (D.R.)

[2] Department of Physical and Inorganic Chemistry, Kaunas University of Technology, Radvilenu 19, LT-50270 Kaunas, Lithuania; andrius.jaskunas@ktu.lt

* Correspondence: kestutis.baltakys@ktu.lt; Tel.: +370-37300163; Fax: +370-7300152

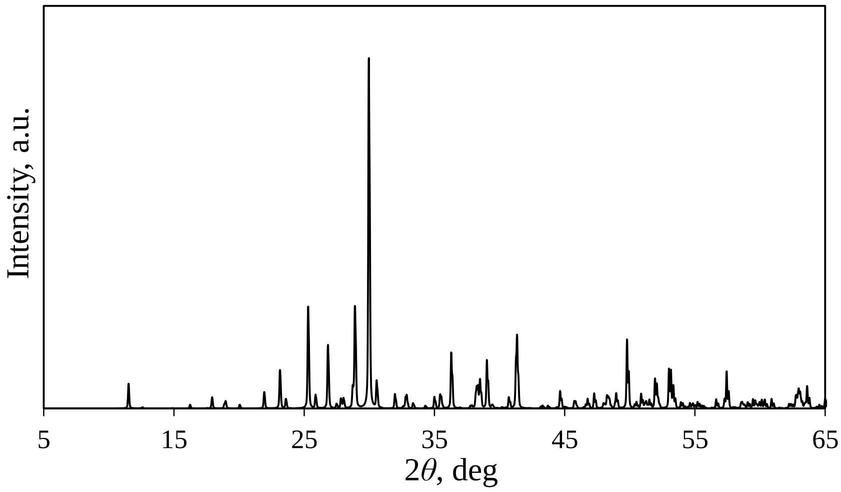

Figure S1. XRD pattern of wollastonite (PDF 00-066-0271).

Table S1. Concentration of Cr^{3+} ions in the liquid medium.

Cr^{3+} Concentration in the Liquid Medium, mg/dm^3		Adsorbed Amount of Cr^{3+}, %	Amount of Cr^{3+} in the Liquid Medium, %
Initial Mixture	After Synthesis		
10,000	0.039	99.99961	0.00039
	0.043	99.99957	0.00043
	0.041	99.99959	0.00041

Table S2. Concentration of NO_3^- ions in the liquid medium.

NO_3^- Concentration in the Liquid Medium, mg/dm^3		Adsorbed Amount of NO_3^-, %	Amount of NO_3^- in the Liquid Medium, %
Initial Mixture	After Synthesis		
17,308	14,116	18.44	81.56
	14,349	17.10	82.90
	13,934	19.49	80.51
